# Reply to the Letter to the Editor entitled: “Cardiology fellow training based on COCATS-4 framework: Limitation and future direction.”

**DOI:** 10.1016/j.ihj.2025.12.004

**Published:** 2025-12-18

**Authors:** Rajesh Vijayvergiya, Atit A. Gawalkar, Mahek Vijayvergiya

**Affiliations:** a Department of Cardiology, Post Graduate Institute of Medical Education & Research, Chandigarh, India; b Department of Cardiology, All India Institute of Medical Sciences, New Delhi, India; c College of Health and Human Development, The Pennsylvania State University, University Park, PA, 16802, USA

We thank Mahajan et al for their interest in our recently published article on cardiology fellow training in India.^1^ Their letter highlights important methodological considerations and provides valuable suggestions that may guide future research in this area.^2^

The concern regarding a relatively small sample size (n = 52) drawn from three tertiary care institutions and the disparity in training curricula across India was acknowledged as a limitation in our original paper. We agree that future studies with larger, multi-centric samples—including peripheral and resource-limited institutions—would improve the generalizability of findings.

Mahajan et al also point out the limitations of relying solely on self-reported competencies.^2^ We acknowledge that self-assessment is inherently subjective and may introduce bias. It is essential to clarify, however, that the COCATS-4 framework itself emphasises the need for periodic, structured, and objective assessments—including direct observation, in-training examinations, procedure logbooks, conference presentations, multisource evaluations, portfolios, simulations, and reflective exercises.^3^ These tools, used in combination, can mitigate the weaknesses of self-report and provide a more holistic evaluation of competency. Importantly, the performance of a certain number of procedures is not synonymous with competence; rather, competence must be judged across knowledge, skills, judgment, and professionalism.

We appreciate the suggestion to employ longitudinal study designs to assess progression over time. While our study was intentionally designed as a cross-sectional assessment at the completion of training, we agree that longitudinal tracking would provide richer insights into competency development and the impact of curriculum interventions. Similarly, the recommendation to integrate qualitative approaches (such as focus group discussions and in-depth interviews) is highly relevant for understanding contextual factors influencing training.

In conclusion, our study aimed to provide the first cross-sectional evaluation of cardiology fellow competencies in India based on the COCATS-4 framework. While limitations such as sample size and reliance on self-report exist, our findings highlight critical training gaps and underscore the need for standardised, competency-based national guidelines. We concur with Mahajan et al that future studies should adopt larger, multi-centric, longitudinal, and mixed-methods approaches to strengthen evidence and inform policy. Ultimately, assessing a cardiology trainee requires a multidimensional approach that integrates objective testing, direct clinical observation, multisource feedback, research activity, and milestone progression—ensuring that a cardiologist is not only technically skilled, but also a safe, ethical, communicative, and adaptive physician.
